# Identification of Potential Biomarkers for Rhegmatogenous Retinal Detachment Associated with Choroidal Detachment by Vitreous iTRAQ-Based Proteomic Profiling

**DOI:** 10.3390/ijms17122052

**Published:** 2016-12-07

**Authors:** Zhifeng Wu, Nannan Ding, Mengxi Yu, Ke Wang, Shasha Luo, Wenjun Zou, Ying Zhou, Biao Yan, Qin Jiang

**Affiliations:** 1 Eye Hospital, Nanjing Medical University, Nanjing 210000, China; zhifengwu2013@126.com; 2 Department of Ophthalmology, Wuxi No. 2 People’s Hospital, Nanjing Medical University, Wuxi 214002, China; dingnn9009@163.com (N.D.); yjmx19860923@126.com (M.Y.); luoshasha403@163.com (S.L.); wendyzwj0805@163.com (W.Z.); habbyzy@sina.com (Y.Z.); 3 Key Laboratory of Nuclear Medicine, Ministry of Health, Jiangsu Key Laboratory of Molecular Nuclear Medicine, Jiangsu Institute of Nuclear Medicine, Wuxi 214063, China; wangke@jsinm.org; 4 The Fourth School of Clinical Medicine, Nanjing Medical University, Nanjing 210000, China; yanbiao1982@hotmail.com

**Keywords:** rhegmatogenous retinal detachment associated with choroidal detachment, rhegmatogenous retinal detachment, proteomics, iTRAQ

## Abstract

Rhegmatogenous retinal detachment associated with choroidal detachment (RRDCD) is a complicated and serious type of rhegmatogenous retinal detachment (RRD). In this study, we identified differentially expressed proteins in the vitreous humors of RRDCD and RRD using isobaric tags for relative and absolute quantitation (iTRAQ) combined with nano-liquid chromatography-electrospray ion trap-mass spectrometry-mass spectrometry (nano-LC-ESI-MS/MS) and bioinformatic analysis. Our result shows that 103 differentially expressed proteins, including 54 up-regulated and 49 down-regulated proteins were identified in RRDCD. Gene ontology (GO) analysis suggested that most of the differentially expressed proteins were extracellular．The Kyoto encyclopedia of genes and genomes (KEGG) pathway analysis suggested that proteins related to complement and coagulation cascades were significantly enriched. iTRAQ-based proteomic profiling reveals that complement and coagulation cascades and inflammation may play important roles in the pathogenesis of RRDCD. This study may provide novel insights into the pathogenesis of RRDCD and offer potential opportunities for the diagnosis and treatment of RRDCD.

## 1. Introduction

Rhegmatogenous retinal detachment associated with choroidal detachment (RRDCD) is a complicated and serious type of rhegmatogenous retinal detachment (RRD). Clinical symptoms of RRDCD include severe uveitis, ocular hypotony, deep anterior chamber, concentric folding, and iridophakodonesis [1,2,3]. RRDCD progresses rapidly and has a poor prognosis because it quickly proceeds to severe proliferative vitreoretinopathy (PVR). The reported incidence of RRDCD in primary RRD is 1.5% to 18.1% in China and 2.0% to 4.5% in Western countries [2,4,5]. RRDCD usually occurs in patients with high myopia, pseudophakia, aphakia, and increased age [5,6]. A recent study has shown that retinal detachment associated with macular hole predisposes one to RRDCD [7]. Visual prognosis after pars planavitrectomy, the conventional treatment for RRDCD, is still unfavorable. The high incidence of PVR after surgery may contribute to the low reattachment rate [8]. To date, most studies have mainly focused on clinical problems. However, the etiology and pathogenesis of RRDCD remain elusive. It is generally believed that hypotony is the initial step of RRDCD [5]. Another hypothesis [3] proposes that intraocular inflammation secondary to retinal detachment plays a pivotal role in the development of choroidal detachment. Given the severe uveitis signs of RRDCD, better prognosis obtained through preoperative application of steroids, as well as our previous study showing that inflammation may participate in the process of RRDCD [9,10], we assume that inflammation has a key role in the occurrence and development of RRDCD.

Proteomics is a post-genomic discipline that studies the proteins of a proteome by various techniques. Since biological activity is executed directly by proteins, studying proteomics in disease or different stages of diseases may shed light on etiopathogenesis and deepen our understanding of diseases, and further help develop better treatment strategies for various diseases. Isobaric tags for relative and absolute quantitation (iTRAQ) is a new technique for protein separation consisting of a unique mass reporter, a mass balancer, and an amine-reactive group [11]. Several features of iTRAQ, such as high through-put, wide range of separation, high accuracy, and repeatability, have led to its widespread use in proteomics.

To date, few studies have studied human vitreous samples from RRDCD patients by proteomic profiling. In this study, we investigated protein profiles of vitreous humor and identified protein biomarkers by iTRAQ combined with nano-liquid chromatography-electrospray ion trap-mass spectrometry-mass spectrometry (nano-LC-ESI-MS/MS) in RRDCD. The objective of this study was to discover differentially expressed proteins and analyze biological processes to improve our understanding of the etiopathogenesis and the poor prognosis of RRDCD.

## 2. Results

### 2.1. Demographics and Clinical Variables

Table 1 shows the demographic and clinical data of the patients enrolled in the study, including 8 RRDCD and 8 RRD patients. There were no significant differences in sex, age, and duration of detachment in the two groups (*p* > 0.05). The RRDCD patients had significantly worse PVR compared to the RRD patients (*p* < 0.001). In addition, the intraocular pressure (IOP) was significantly lower in RRDCD than in RRD patients (*p* < 0.005).

### 2.2. Protein Concentration

Protein concentration was 3.31 ± 1.36 µg/µL in the RRDCD group and 2.21 ± 0.35 µg/µL in the RRD group. The results show that protein concentration in RRDCD patients was significantly higher than in RRD patients (*p* = 0.043).

### 2.3. Integrated Proteome Information

**Figure 1:**
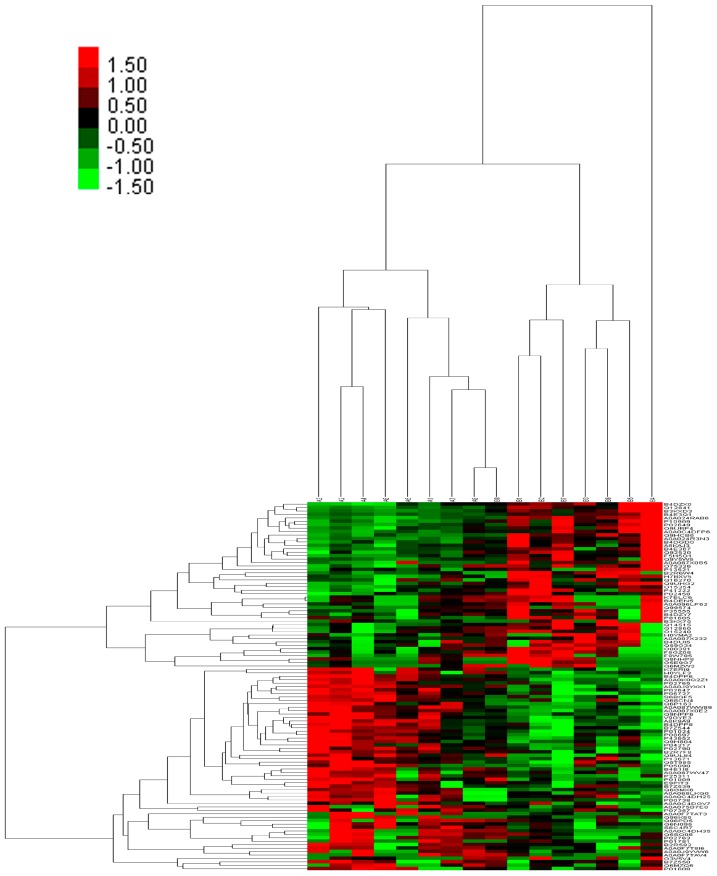
Heat map of differentially expressed proteins between rhegmatogenous retinal detachment (RRD) and rhegmatogenous retinal detachment associated with choroidal detachment (RRDCD). The color scale shown at the top illustrates the protein expression fold change of all the samples. Red represents up-regulated protein expression of RRD compared to RRDCD, whereas green represents down-regulated protein expression of RRD compared to RRDCD.

A total of 2510 unique peptides and 750 proteins were identified by iTRAQ-mass spectrometry. One hundred and three differentially expressed proteins were identified (fold change > 1.2 and *p* value < 0.05), including 54 up-regulated and 49 down-regulated proteins in the RRDCD group compared to the RRD group (Table A1 and Table A2). The hierarchical clustering heat map of the differentially expressed proteins is shown in Figure 1.

### 2.4. Gene Ontology Annotation of Differentially Expressed Proteins

**Figure 2:**
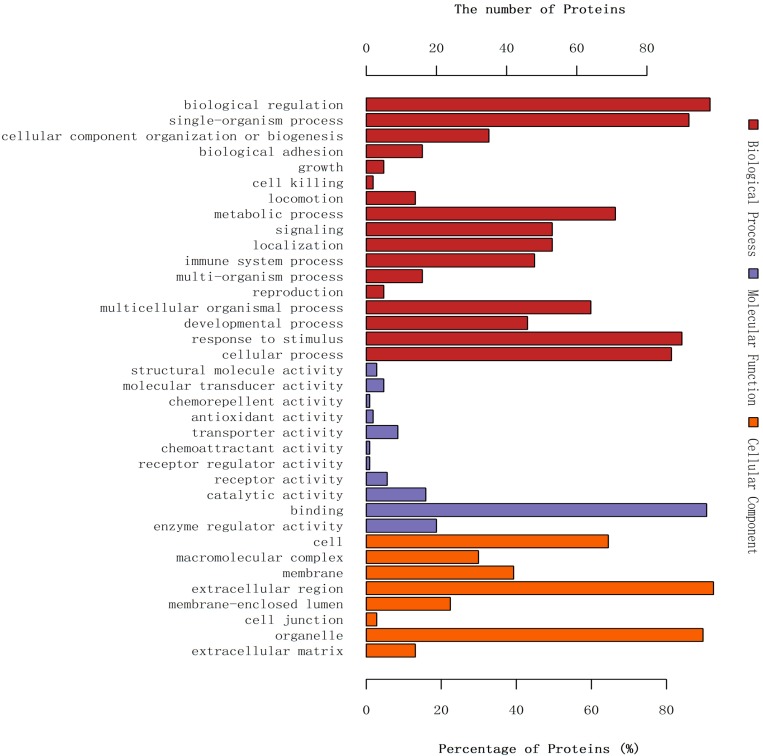
Gene ontology (GO) annotation of differentially expressed proteins. Most of the proteins of differential abundance analyzed for biological process, molecular function, and cellular component were biological regulation, binding, and extracellular region, respectively.

Gene ontology (GO) is an international standardized gene function classification system, offering an updated controlled vocabulary to describe the properties of gene and gene products [12]. As shown in Figure 2, when analyzed for biological process (BP) the differentially expressed proteins were primarily involved in biological regulation (98, 12.3%), single-organism process (97, 11.5%), and response to stimulus (90, 11.3%). When analyzed for molecular function (MF) these proteins were mainly involved in binding (97, 59.9%), enzyme regulator activity (20, 12.3%), and catalytic activity (17, 10.5%). When analyzed for cellular component (CC), these proteins were primarily categorized as extracellular space (99, 26.1%), organelle (96, 25.3%) and cell (69, 18.2%).

**Figure 3:**
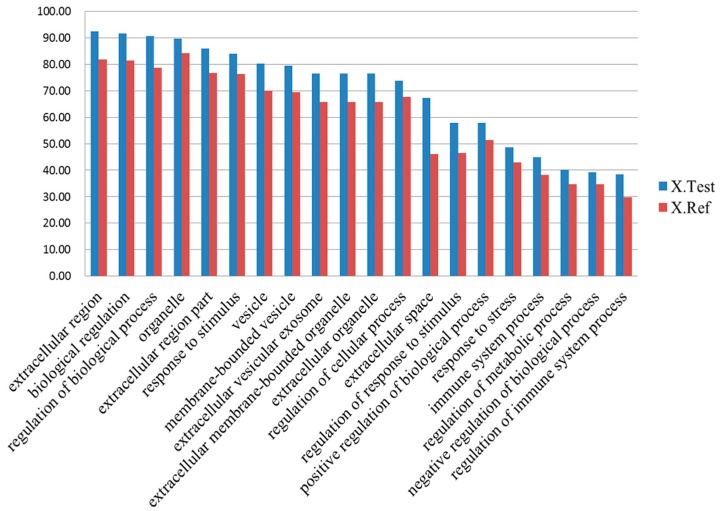
Gene ontology (GO) enrichment analysis. The GO terms of most differentially expressed proteins were mainly enriched in extracellular space, followed by biological regulation. Regulation of biological process. The Y axis represent the proportion of differentially expressed proteins. X. Test represents the proportion of differentially expressed proteins related to the GO term in all differentially proteins. X. Ref represents the proportion of proteins related to the GO term in all identified proteins.

GO enrichment analysis of the differentially expressed proteins showed that the GO terms of most differentially expressed proteins were enriched in extracellular space (*p* = 9.29 × 10^−7^), followed by biological regulation (*p* = 0.001) and regulation of biological process (*p* = 0.0003, Figure 3).

### 2.5. Kyoto Encyclopedia of Genes and Genomes Analysis

**Figure 4:**
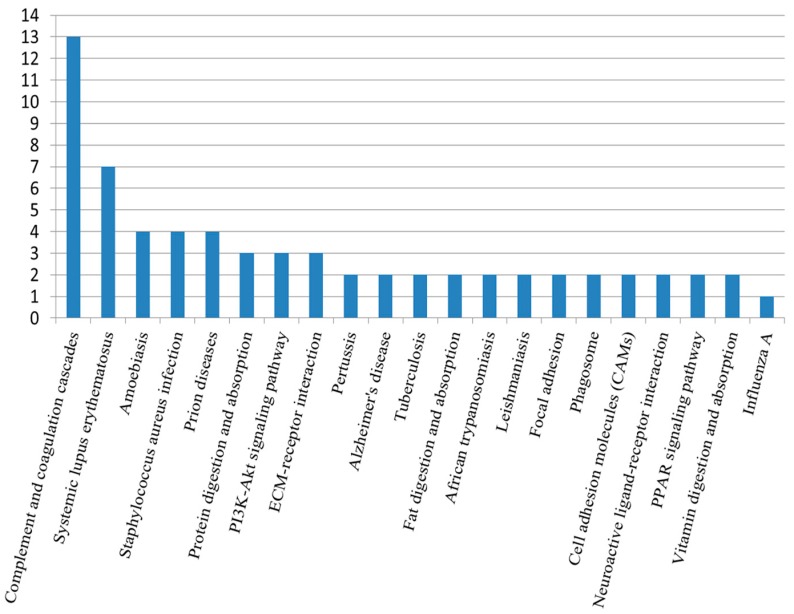
Kyoto encyclopedia of genes and genomes (KEGG) pathway anlaysis of differentially expressed proteins. The complement and coagulation pathway was enriched in the majority of the differentially expressed proteins. The second highly enriched pathway was the systemic lupus erythematosus pathway. The vertical bars represent the number of differentially expressed proteins.

**Figure 5:**
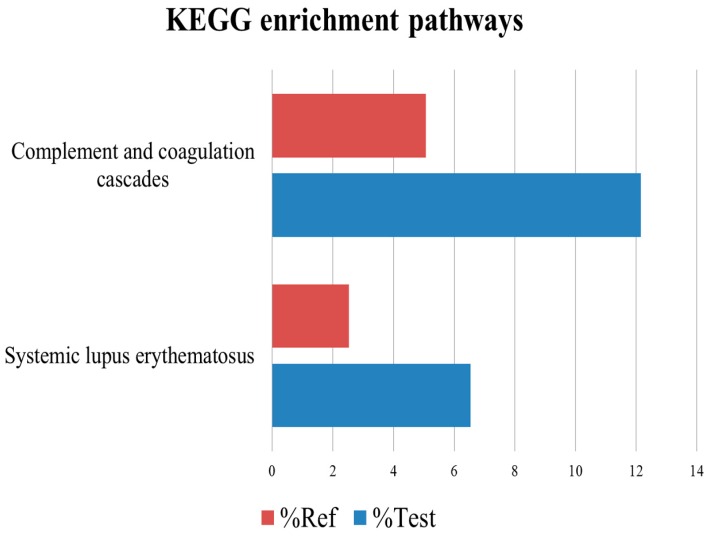
Enrichment of differentially expressed proteins in Kyoto encyclopedia of genes and genomes (KEGG) pathways. The significant enriched pathways were the complement and coagulation pathway and systemic lupus erythematosus. %Test represents the number of differentially expressed proteins related to the KEGG pathways in all differentially proteins. %Ref represents the number of proteins related to the KEGG pathways in all identified proteins. The vertical bars represent the number of differentially expressed proteins.

**Figure 6:**
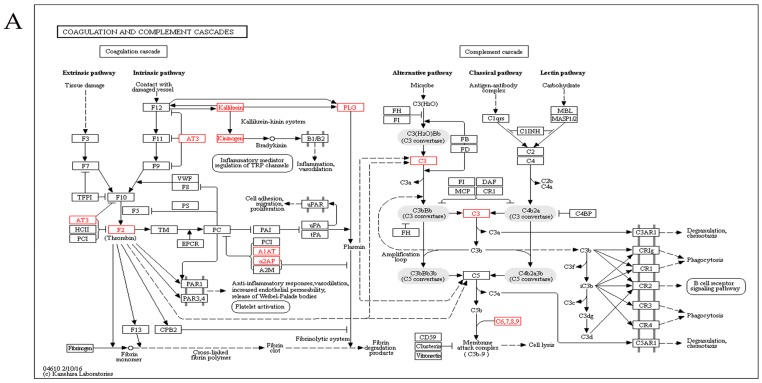

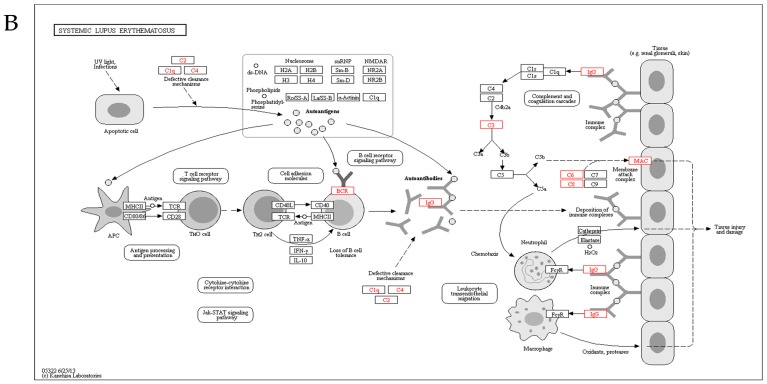
Kyoto encyclopedia of genes and genomes (KEGG) pathway enrichment analysis maps of the complement and coagulation pathway (**A**) and systemic lupus erythematosus (**B**). The proteins in red frames are differentially expressed proteins identified in this study. The box represents proteins, the solid arrow means activation, the dash arrow means indirect activation.

Using the Kyoto encyclopedia of genes and genomes (KEGG) pathway database, the differentially expressed proteins were classified into 65 pathways, the top 21 of which are shown in Figure 4. The complement and coagulation pathway was the top pathway, followed by the systemic lupus erythematosus (SLE) pathway. KEGG analysis showed that the significantly enriched pathways included the complement and coagulation and SLE pathways (Figure 5). The KEGG maps are shown in Figure 6.

### 2.6. Protein Verification by Western Blotting Analysis

**Figure 7:**
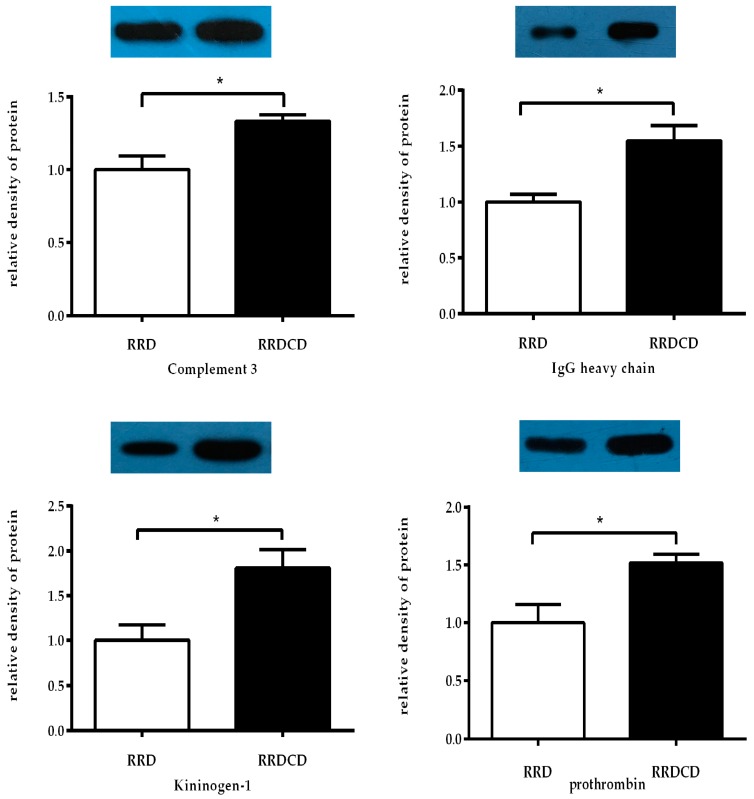
Western blotting analyses of 4 differentially expressed proteins. Changes in protein abundance shown by Western analysis and quantification of the proteins were highly consistent with the proteomic data of patients. * Represents *p* < 0.05.

C3, prothrombin, kininogen-1, and IgG heavy chain were randomly chosen and detected in all RRD (*n* = 8) and RRDCD (*n* = 8) vitreous samples by Western analysis. Changes in protein abundance shown by Western analysis and quantification of the proteins were highly consistent with the proteomic data of patients (Figure 7).

## 3. Discussion

This study investigated the proteomic profiles of RRDCD vitreous humor using the iTRAQ and nano-LC-ESI-MS/MS techniques. We identified 103 differentially expressed proteins, including 54 up-regulated and 49 down-regulated proteins, in the RRDCD samples compared to the RRD samples. We randomly selected four proteins for validation by Western blot analysis. The changes in protein abundance were highly consistent with the proteomic data.

According to GO annotation analysis, the differentially expressed proteins in the RRDCD samples were primarily assigned to biological regulation, binding, and extracellular region when analyzed for BP, MF and CC, respectively. GO enrichment analysis showed that most of the differentially proteins were enriched in the extracellular space. These proteins may be secreted from the neighboring tissues or derived from the broken blood-retinal barrier (BRB). The complement, coagulation and SLE pathways were significantly enriched in KEGG pathway analysis.

Most up-regulated proteins in the vitreous humor of the RRDCD group, compared to those in the RRD group, were plasma proteins. This might be caused by the leakage from the suprachoroidal space or retinal vessels into the vitreous. These proteins might not be inductive factors for RRDCD. It is possible that the level of protein concentration is useful for diagnosis or predictions of RRDCD.

This study identified highly abundant proteins associated with inflammation in RRDCD, such as α-1-antitrypsin, immunoglobulin chains and complement proteins (C1r, C3, C6 and C8). α-1-antitrypsin is a member of the serpin superfamily. Its typical function is inhibiting serine protease like neutrophil elastase, proteinase 3, trypsin, kallikreins and so on [13]. α-1-antitrypsin is an acute-phase response prtein and involves in reducing production of inflammatory mediators, blocking inflammatory cells to modulates inflammatory responses [14]. The previous studies show that complement components and immunoglobulins existed in epiretinal membranes of PVR [15,16]. The immunoglobulin heavy and light chains were significantly up-regulated in the vitreous of RRDCD, indicating that the immune reaction or BRB breakdown in RRDCD was more serious than in RRD.

The complement system is an important constituent of human innate immunity and a bridge to adaptive immunity [17]. C3a is product of C3 activation, also known as anaphylatoxin. Activation of the G protein coupled receptors (C3aR) by C3a promotes degranulation in mast cells and basophils and release of histamine and other mediators, finally resulting in inflammatory hyperemia and edema [18,19,20]. C3a recruits neutrophils, macrophages and other inflammatory cells and induces vasodilation and inflammatory mediator release [18,19,20]. High expression of C3 in the RRDCD vitreous humor can result in intraocular inflammation and destruction of ocular tissues like retina and vessels. This process may lead to RRDCD pathophysiological processes. C6 and C8 participate in the formation of the membrane attack complex (MAC) C5–C9, and subsequently lead to cell lysis [21]. Cashman and colleagues found that mice injected with C3-expressing adenovirus significantly increased vascular permeability, RPE atrophy, endothelial cell proliferation, and migration [18]. Our results support this previous study suggesting a role of complement in RRDCD. The complement components may cause increased retinal vascular permeability, retinal damage, cell proliferation and inflammation, which in turn to promote RRDCD and contribute to PVR and poor visual prognosis.

In this study, coagulation proteins, such as prothrombin, α-1-antitrypsin, α-2-antiplasmin and plasminogen, were significantly up-regulated in the RRDCD vitreous humor. This may be due to the penetration of plasma proteins due to broken BRB and dysfunction of retinal vessels. Blood coagulation is a process in which successive activation of coagulation factors triggers a chain reaction, resulting in fibrin clot formation. The activated coagulation cascade has a positive feedback effect [22,23]. Additionally, there is a reciprocal relationship between coagulation and inflammation [24,25]. Thrombin, which is activated from prothrombin and was identified in this study, is a serine protease that mediates a strong inflammatory response [25]. Up-regulated coagulation proteins may further aggravate ocular inflammation. The coagulation cascade also has a reciprocal relationship with the complement system. The dysregulation of one system can escalate the activation of both. A previous study revealed that plasminogen mutually and prominently activates and coexists with complement in many chronic inflammation diseases [26]. Coagulation cascade components have been found in vitreous humor by some proteomic studies for other vitreoretinal diseases [27,28], but significant enrichment in RRDCD has not been reported. The significantly enriched coagulation components in RRDCD compared to RRD indicate that increased coagulation activity plays a role in the development of RRDCD. This study showed that kininogen-1 and plasma kallikrein B, the components of the kallikrein-kinin system, were up-regulated in the vitreous humor of RRDCD compared to RRD. The kallikrein-kinin system plays important roles in many physiological and pathological processes such as regulation of vasomotor tone, blood coagulation, fibrinolysis process, inflammation, and electrolyte balance [29,30,31,32]. Plasma kallikrein and high molecular weight kininogen (HMWK) circulate as a complex that binds to plasma membrane of the endothelial cells, therefore, we can assume that the complex also exists in retinal vessels. A previous study has reported that high levels of bradykinin B1 and B2 receptors are expressed in all of the layers of human retina and the vascular endothelial cells [33]. This suggests that the plasma kallikrein/kininogen might participate in the regulation of retinal vessel functions. It has been reported that intraocular hemorrhage increases retinal vascular permeability and leukocyte stasis induced by plasma kallikrein [34]. Another study has shown that intravitreal injection of plasma kallikrein in rats increases retinal vascular permeability [35]. The components of the kinin-kallikrein system probably cross the BRB into the vitreous fluid and enhance vascular permeability. Additionally, plasma kallikrein/kininogen might interact with coagulation components and inflammatory factors, finally promoting PVR and RRDCD disease progression.

Some other proteins, like apolipoprotein, serum albumin, hemopexin, and retinol binding protein 4 (RBP4), were up-regulated in the vitreous of RRDCD. These proteins are important for maintaining cell activities and transport functions. Recently, a few studies indicated that increased RBP4 in blood may promote retinal dysfunction [34,36,37].

Most down-regulated proteins in the vitreous humor of the RRDCD group compared to those in the RRD group were development related proteins. This may indicate that the pathogenesis of RRDCD is more severe and complicated compared to RRD. Prostaglandin-H2 d-isomerase (PTGDS) catalyzes change of prostaglandin H2 to prostaglandin D2 in the arachidonic acid (AA) metabolic pathway. In our study, PTGDS was down-regulated in the vitreous of RRDCD, consistent with the results in our previous metabolomics study of down-regulated AA in the vitreous of RRDCD patients [10].

Although proteomic techniques were already developed and can obtain accurate data to provide additional insights into the development of RRDCD, there were several limitations in this study. First, the number of vitreous samples was relatively small. RRDCD is a disease with low prevalence. In our department, only 13 samples of RRDCD were collected between September 2014 and September 2015, after RRDCD patients administered with preoperative steroids and patients with recurrent RRDCD, secondary RRDCD and comorbid diseases were excluded. However, there was statistically significant difference between the two groups in our study, and the results of WB were consistent with the proteomic data, suggesting that the results are credible; Second, due to the limited number of reliable and constantly updated reference databases for protein identification, our interpretation of the results was limited; Lastly, it is difficult to know what to make of protein interaction networks in a fluid like vitreous humor. Our data offer another perspective to understand the function of retina and choroid in process of RRDCD. The results and conclusions of this study still need to be improved by expanding the samples' size and verified by blood samples in the future research.

## 4. Materials and Methods

### 4.1. Patients

Eight primary RRD and 8 primary RRDCD patients, undergoing pars plana vitrectomy between September 2014 and September 2015 at Nanjing Medical University Affiliated Wuxi Second Hospital (Wuxi, China) were enrolled in this study. The exclusion criteria included diabetic retinopathy, intraocular inflammation, systemic disease such as hypertension, diabetic, previous intraocular surgery and intravitreal injection with steroid or anti-vascular endothelial growth factor. All subjects received comprehensive ophthalmic examination before operation by two experienced doctors. The grade of Proliferative vitreoretinopathy (PVR) was recorded on the basis of the classification of the Retina Society Terminology Committee [38]. The symptom duration was recorded in days and the extents of retinal detachment were recorded in quadrants.

### 4.2. Ethics Statement

The study was followed the principles of Helsinki Declaration and was approved by the ethics committee of Nanjing Medical University affiliated Wuxi NO.2 Hospital (Wuxi, China. Identification code: WXEYLL256. 29 August 2014). All subjects in this study signed a copy of informed consent prior to participation.

### 4.3. Sample Preparation

Approximately 0.5–1.0 mL vitreous samples were collected using a 5 mL sterile syringe when the 25-gauge trocar was inserted into the vitreous cavity before active infusion. Undiluted vitreous fluid was transferred into 1.5 mL Eppendorf Tubes immediately and centrifuged at 9398× *g* for 10 min at 4 °C. The supernatants were aliquoted and stored at −80 °C until analysis. The samples were thawed and mixed with 800 µL SDT lysis buffer (4% SDS, 100 mM Tris-HCl, 1 mM DTT, and pH 7.6), followed by sonication. After being incubated in a 95 °C water bath for 15 min, the samples were centrifuged at 14,000× *g* for 40 min. The protein concentration of the supernatants was determined by Bicinchoninic acid protein assay kit (Beyotime, P0012, Nantong, China).

### 4.4. Protein Digestion and iTRAQ Labeling

Filter aided proteome preparation (FASP) was performed as previously described for proteins digestion [39]. The finally filtrates were collected and the peptides were quantified at 280 nm. According to the manufacturer’s instructions, 100 µg samples from each group were labeled by iTRAQ Reagent-4plex multiplex kit (AB SCIEX, Foster City, CA, USA). Proteins from RRDCD group and RRD group were labeled with reagent 114 and reagent 116 respectively. Blank control group were labeled with reagent 117.

### 4.5. Strong Cationic-Exchange (SCX) Chromatography Separation

The labeled peptides of samples were mixed and acidified with 2 mL of buffer A (10 mM KH_2_PO_4_, pH 3.0, and 25% (*v*/*v*) acetonitrile (ACN)). The mixed peptides were then loaded onto a Polysulfoethyl™ (Poly-LC Inc., Columbia, MD, USA) column (4.6 × 100 mm, 5 µm, 200 Å). The peptides were eluted at a flow rate of 1000 µL/min, setting the gradient at 0%–100% of buffer B (10 mM KH_2_PO_4_, 500 mM KCl, 25% ACN, and pH 3.0). Absorbance at 214 nm was monitored during elution, and the fractions were collected every minute. Approximately 30 fractions were collected and combined into 10 pools and desalted on C18 Cartridges.

### 4.6. Nano-LC-ESI-MS/MS Analysis

The fractions were analyzed using Q Exactive MS equipped with Easy nLC (Thermo Finnigan, San Jose, CA, USA). The chromatographic columns were balanced with buffer A (0.1% formic acid). Peptide mixture (2 µg) was loaded via an auto-sampler into a Thermo scientific EASY column (2 cm × 100 µm, 5 µm-C18) and separated by analytical columns (75 µm × 100 mm, 3 µm-C18) at a flow rate of 250 nL/min. The linear gradient was set as 0%–100% buffer B (10 mM KH_2_PO_4_, 500 mM KCl, 25% ACN, and pH 3.0) for 240 min. After separation of nano-HPLC, samples were analyzed by the Q-Exactive mass spectrometer in a positive ion mode. The parent Ion Scan range set as 300–1800 *m*/*z*. 10 MS2 scans were collected after every full scan to obtain the mass-to-charge ratio of peptides/peptides fragments.

### 4.7. Protein Identification and Quantification

The raw dates from Q Exactive were submitted to Mascot search software (version 2.2.1; Matrix Science, London, UK) and Proteome Discoverer 1.4 (Thermo Electron, San Jose, CA, USA) for date filtering and proteins identification. Furthered analysis was performed according to false discovery rate (FDR) ≤0.01 using Proteome Discoverer 1.4. Protein identification was supported by at least one unique peptide identified using the Peptide Prophet algorithm. Proteins with fold change >1.2 were retained. Hierarchical clustering analysis was performed by MeV 4.7 software, and Pearson’s correlation analysis was used for the distance matrix and the Ward’s linkage.

### 4.8. Bioinformatic Analysis

Proteins/peptides sequences were imported into Blast2GO for GO annotation and enrichment [12]. The differentially expressed proteins were then blasted against KEGG GENES to retrieve their KEGG Orthology (KOs) and were mapped to KEGG pathways [40]. Fisher’s exact test was carried out to evaluate the enrichment of GO terms and KEGG pathways of differentially expressed proteins against the annotation of all identified proteins.

### 4.9. Western Blotting

Western blot analyses were performed to validate the proteomic data on some randomly chosen differentially expressed proteins. Briefly, equal amounts of proteins (20 µg) for each sample were loaded on a 10% sodium dodecyl sulfate polyacrylamide gel. After blocking with 5% bovine serum albumin, the membranes were incubated with monoclonal antibodies of C3 (21337-1-AP; Proteintech, Chicago, IL, USA), prothrombin (ab109087; Abcam, Cambridge, MA, USA), kininogen-1 (ab175386; Abcam), and IgG heavy chain (16402-1-AP; Proteintech), followed by incubation with secondary antibodies (sc-2004; Santa Cruz, CA, USA). Protein bands were visualized using the ECL assay kit (P0018; Beyotime, Nantong, China). The quantification of the proteins was done using Image J software (http://rsb.info.nih.gov/ij/).

## 5. Conclusions

We performed a proteomic study using iTRAQ-nano-LC-ESI-MS/MS techniques and bioinformatic analyses to identify differentially expressed proteins between RRDCD and RRD. Based on our findings, the proteins of complement and coagulation cascades, the kallikrein-kinin system, and inflammation may play a crucial role in the course of RRDCD. This study provides insights into the development of RRDCD and warrants further studies in the future.

## Figures and Tables

**Table 1:** Clinical characteristics of the study population.

Clinical Characteristics	RRDCD	RRD	*p* Values
	*N* = 8	*N* = 8	
Sex, *n* (%)			
Males	3 (37.5)	3 (37.5)	1.0
Females	5 (62.5)	5 (62.5)	
Age, *y*			
Median (range)	60 (46–65)	59.5 (39–65)	0.959
Duration of detachments, *d*			
Median (range)	8.5 (5–13)	13 (7–20)	0.065
PVR grade			
Mild, A, B	1	8	0.001
Heavy, C, D	7	0	
IOP, mmHg			
Median (range)	7.75 (6.8–13.4)	14 (11.9–17.2)	0.002

*p* Value was calculated by Mann–Whitney *U* test or *Chi-square* test. RRDCD: Rhegmatogenous retinal detachment associated with choroidal detachment; RRD: Rhegmatogenous retinal detachment; PVR: Proliferative vitreoretinopathy; A–D: The grade of PVR; IOP: Intraocular pressure; N: Numbers; Y: Years; D: Days.

## Abbreviations

AA	Arachidonic acid
BP	Biological process
BRB	Blood-retinal barrier
CC	Cellular component
GO	Gene ontology
iTRAQ	Isobaric tags for relative and absolute quantitation
KEGG	Kyoto encyclopedia of genes and genomes
MAC	Membrane attack complex
MF	Molecular function
Nano-LC-ESI-MS/MS	Nano-liquid chromatography-electrospray ion trap-mass spectrometry-mass spectrometry
PTGDS	Prostaglandin-H2 d-isomerase
PVR	Proliferative vitreoretinopathy
RRD	Rhegmatogenous retinal detachment
RRDCD	Rhegmatogenous retinal detachment associated with choroidal detachment
SLE	Systemic lupus erythematosus

## Appendix A

**Table A1:** 54 up-regulated proteins in vitreous humor of rhegmatogenous retinal detachment associated with choroidal detachment (RRDCD) compared to rhegmatogenous retinal detachment (RRD) identified from iTRAQ analysis.

Accession	Description	Coverage	Unique Peptides	Peptides	Fold Change	*t* Test *p* Value
K7ERI9	Apolipoprotein C-I	23.38	3	3	2.72074	6.32 × 10^−5^
A0A087X0E2	Serum amyloid A protein	22.95	3	3	2.548367	0.000442
A0A0F7TAV4	IGHV5-51 protein (Fragment)	16.36	2	2	1.935654	0.032955
P00738	Haptoglobin	30.05	15	15	1.925942	0.021916
Q6P163	APOC2 protein	25	2	2	1.803961	0.0011
Q9NPP6	Immunoglobulin heavy chain variant	17.79	2	9	1.799359	0.001327
A0A075B7E8	Protein IGHV3OR16-13	11.97	2	2	1.789869	0.010504
H0YLF3	β-2-Microglobulin (Fragment)	28.17	2	2	1.782909	0.00658
P05090	Apolipoprotein D	30.16	6	6	1.763858	0.045205
B4DPP6	Serum albumin	53.88	5	49	1.748857	0.000246
V9GYE3	Apolipoprotein A-II	42.31	5	5	1.740055	0.000297
A8K9A9	Kallikrein B, plasma	12.54	8	8	1.719625	0.000228
P02790	Hemopexin	19.26	12	12	1.622425	0.000326
Q9H804	Completement C1R	7.31	2	3	1.580432	0.006066
P02763	α-1-Acid glycoprotein 1	16.42	2	6	1.572967	0.009053
P02647	Apolipoprotein A-I	46.44	17	17	1.56536	0.00079
A0A0K0Q2Z1	Serpin peptidase inhibitor clade C member 1	19.83	13	13	1.562564	0.000278
B7Z544	Inter-α-trypsin inhibitor heavy chain H4	11.71	11	11	1.546281	0.003001
Q96PD5	*N*-acetylmuramoyl-l-alanine amidase	7.29	3	3	1.545521	0.00139
B4DPP8	Kininogen-1	30.84	17	17	1.48514	0.000647
B4E1I8	Leucine-rich α-2-glycoprotein	15.15	6	6	1.458316	0.006872
E9PIT3	Prothrombin	13.04	8	8	1.455448	0.013628
P43652	Afamin	25.04	17	17	1.453959	0.003423
P08697	α-2-Antiplasmin	12.83	7	7	1.450495	0.008491
B2R582	C-type lectin domain family 3, member B	21.78	4	4	1.447796	0.002065
P06727	Apolipoprotein A-IV	42.17	21	21	1.44284	0.000649
Q5SQ08	Complement component C8 γ chain	11.59	2	2	1.431101	0.018442
Q9UL84	Myosin-reactive immunoglobulin heavy chain variable region	14.75	2	2	1.387229	0.017727
P04217	α-1B-Glycoprotein	13.74	7	7	1.372869	0.014165
B7Z539	Inter-α-trypsin inhibitor heavy chain H1	7.91	7	7	1.363604	0.027945
B2R7F8	Plasminogen	24.2	22	22	1.360977	0.007811
A0A0C4DGV7	Retinol-binding protein 4	14.07	4	4	1.359868	0.04222
P01024	Complement C3	24.95	54	54	1.341747	0.002478
P02765	α-2-HS-glycoprotein	26.16	10	10	1.333365	0.00478
B7Z550	Complement component 8, β polypeptide	7.56	4	4	1.329365	0.007697
P07357	Complement component C8 α chain	11.47	7	7	1.320078	0.04949
Q5T985	Inter-α-trypsin inhibitor heavy chain H2	15.51	17	17	1.310572	0.030167
P25311	Zinc-α-2-glycoprotein	30.2	10	10	1.307167	0.031312
P01009	α-1-Antitrypsin	27.51	14	14	1.303075	0.020778
P13671	Component C6	6.64	7	7	1.224111	0.03757

Uniprot accession numbers that can be found on www.uniprot.org; Coverage: The percentage of the protein sequence covered by identified peptides. Unique Peptides: The number of peptide sequences unique to a protein group. Peptides: The number of distinct peptide sequences in the protein group. Fold change: The quantity changes of protein abundance between the two groups.

**Table A2:** 49 down-regulated proteins in vitreous humor of RRDCD compared to RRD identified from iTRAQ analysis.

Accession	Description	Coverage	Unique Peptides	Peptides	Fold Change	*t* Test *p* Value
Q16270	Insulin-like growth factor-binding protein 7	28.72	7	7	0.795347	0.048896
H7BXV5	Collagen α-1(XVIII) chain	6.84	4	4	0.72897	0.007309
B4DEN5	*N*-acetyllactosaminide β-1,3-*N*-acetylglucosaminyltransferase	14.71	6	6	0.697059	0.007153
A8KAJ3	EGF-containing fibulin-like extracellular matrix protein 1	22.31	10	10	0.694626	0.020864
O75326	Semaphorin-7A	14.56	9	9	0.688332	0.008646
Q12860	Contactin-1	8.35	8	8	0.687135	0.010164
P41222	Prostaglandin-H2 d-isomerase	17.89	7	7	0.673721	0.012421
Q9UHG2	ProSAAS	15	3	3	0.67195	0.006702
Q8NHP8	Putative phospholipase B-like 2	3.57	2	2	0.669177	0.017856
Q92520	Protein FAM3C	24.23	6	6	0.668184	0.00515
O15240	Neurosecretory protein VGF	14.15	8	8	0.661762	0.010221
B4DZY7	Growth-arrest-specific protein 6	5.72	3	3	0.650294	0.033296
B4DUI5	Triosephosphate isomerase	25.82	5	5	0.642348	0.009858
A0A0C4DFP6	Cartilage acidic protein 1	9.73	7	7	0.636797	0.005166
P10909	Clusterin	25.84	6	14	0.629457	0.000292
B4DZX0	Nucleobindin-1	14.51	7	7	0.627294	0.000278
A0A087X0S5	Collagen α-1(VI) chain	8.09	8	8	0.617461	0.013995
Q9UBP4	Dickkopf-related protein 3	18.57	6	6	0.603169	0.004542
O00391	Sulfhydryl oxidase 1	9.37	7	7	0.598255	0.003939
P35555	Fibrillin-1	1.99	7	7	0.597125	0.005083
F8W785	Golgi integral membrane protein 4	6.59	4	4	0.592385	0.022707
B4DGD0	Amyloid β A4 protein	11.34	8	9	0.586216	0.009153
F5GZ08	Amyloid-like protein 1	15.71	7	10	0.582642	0.011373
O15354	Prosaposin receptor	2.77	2	2	0.567766	0.016087
P02458	Collagen α-1(II) chain	3.36	5	5	0.564508	0.025358
A0A087X232	Complement C1s subcomponent	6.45	5	5	0.539272	0.021601
A0A096LP62	Inter-α-trypsin inhibitor heavy chain H5	7.28	5	5	0.53903	0.024978
P81605	Dermcidin	27.27	4	4	0.537086	0.029414
F5H5G1	Limbic system-associated membrane protein	6.6	2	2	0.528697	0.007773
Q12841	Follistatin-related protein 1	28.9	9	9	0.495666	0.000459
Q14515	SPARC-like protein 1	11.6	7	7	0.494821	0.001398
Q99574	Neuroserpin	7.8	4	4	0.493937	0.026239
Q6MZW2	Follistatin-related protein 4	4.51	2	3	0.485352	0.028432
P02649	Apolipoprotein E	63.72	20	22	0.480489	0.00203
A0A024RAB6	Heparan sulfate proteoglycan 2 (Perlecan)	3.89	17	17	0.479996	6.82E-05
P13521	Secretogranin-2	6.97	4	4	0.470064	0.028148
B4E3Q1	Calsyntenin-1	10.71	13	13	0.440009	0.008494
B3KX75	Neural cell adhesion molecule L1-like protein	3.14	4	4	0.431103	0.04737
Q9HCB6	Spondin-1	11.77	10	10	0.412771	0.000529
B3KXD3	Carboxypeptidase E	20.43	10	10	0.376381	0.001059
A0A024R3N3	Amyloid β (A4)-like protein 2	12.68	8	9	0.37116	0.001787
B4E367	Plexin domain-containing protein 2	3.69	2	2	0.363499	0.006849
B2R6W4	Frizzled-related protein (FRZB)	11.69	4	4	0.291076	0.000102
Q9Y5W5	Wnt inhibitory factor 1	10.82	4	4	0.290436	0.009571

Uniprot accession numbers that can be found on www.uniprot.org; Coverage: The percentage of the protein sequence covered by identified peptides. Unique Peptides: The number of peptide sequences unique to a protein group. Peptides: The number of distinct peptide sequences in the protein group. Fold change: The quantity changes of protein abundance between the two groups.
